# Investigation of Key Signaling Pathways Associating miR-204 and Common Retinopathies

**DOI:** 10.1155/2021/5568113

**Published:** 2021-10-04

**Authors:** Ahmad Bereimipour, Leila Satarian, Sara Taleahmad

**Affiliations:** ^1^Department of Stem Cells and Developmental Biology, Cell Science Research Center, Royan Institute for Stem Cell Biology and Technology, ACECR, Tehran, Iran; ^2^Faculty of Sciences and Advanced Technologies in Biology, University of Science and Culture, Tehran, Iran; ^3^Department of Molecular Systems Biology, Cell Science Research Center, Royan Institute for Stem Cell Biology and Technology, ACECR, Tehran, Iran

## Abstract

MicroRNAs are a large group of small noncoding RNAs that work in multiple cellular pathways. miR-204, as one of the key axes in the development, maintenance, and pathogenesis of the retina, plays several roles by modulating its target genes. This study was aimed at evaluating the target genes of miR-204 involved in the development and progression of common retinopathies such as glaucoma, retinoblastoma, and age-related macular degeneration. In this study, three datasets related to retinopathies (GSE50195, GSE27276, and GSE97508) were selected from Gene Expression Omnibus. miR-204 target genes were isolated from TargeScan. The shares between retinopathy and miR-204 target genes were then categorized. Using Enrichr and STRING, we highlighted the signaling pathways and the relationships between the proteins. SHC1 events in ERBB2, adherent junction's interactions, NGF signaling via TRKA from the plasma membrane, IRF3-mediated activation of type 1 IFN, pathways in upregulated genes and G0 and early G1, RORA-activated gene expression, PERK-regulated gene expression, adherent junction's interactions, and CREB phosphorylation pathways in downregulated genes were identified in glaucoma, retinoblastoma, and age-related macular degeneration. WEE1, SMC2, HMGB1, RRM2, and POLA1 proteins were also observed to be involved in the progression and invasion of retinoblastoma; SLC24A2 and DTX4 in age-related macular degeneration; and EPHB6, EFNB3, and SHC1 in glaucoma. Continuous bioinformatics analysis has shown that miR-204 has a significant presence and expression in retinal tissue, and approximately 293 genes are controlled and regulated by miR-204 in this tissue; also, target genes of miR-204 have the potential to develop various retinopathies; thus, a study of related target genes can provide appropriate treatment strategies in the future.

## 1. Introduction

The retina is a specific layer of the eye with various layers, each of them including special cells. Neurological disorders have been observed in various retinopathies related to disturbances in each of these retinal cells and their networks [1].

Glaucoma is one of the most common retinopathies that occur due to disruption of retinal ganglion cells [2]. Age-related macular degeneration (AMD) is age-related retinopathy due to the retinal pigment epithelium dysfunction [3]. Retinoblastoma is also one of the most common intraocular cancers in children, with blue cone photoreceptor cells being the disease's source [4]. These three retinopathies affect many people each year and lead to blindness or enucleation of patients' eyes if not prevented by timely treatment.

MicroRNAs (miRNAs) are small noncoding RNAs of between 20 and 22 nucleotides and are made by RNA polymerase 2 [5]. miRNAs act as very important regulatory elements in various intracellular and extracellular pathways of different processes including cell adhesion. miRNAs regulate the expression of several genes by transient inhibitory mechanisms [6, 7]. Therefore, dysfunction of miRNAs may have many side effects on cell activity and may eventually lead to many diseases.

So far, several miRNAs have been found in the eye and retina layers with specific roles; for instance, miR-204 has been shown to be actively involved in nerve cell development, and it is highly expressed in the formation of the lens [8, 9]. Conte et al. showed that miR-204 plays an important role in the development of the retina and optic cup by different expression patterns [10]. The crosstalk between miR-204 and meis2 is a key part of controlling eye morphology and differentiation [10].

To date, various bioinformatics studies have identified miRNA involvement in different processes such as the development and maintenance of retinal diseases [11]. Several studies have examined the association between miRNAs and their target genes in some retinopathies. It has been reported that on high-altitude retinopathy, miR-3177-3p, miR-369-3p, miR-603, and *FOS*, *IL10*, and *IL-7R* play a role in the progression of the disease [11]. Studies have also been performed on diabetic retinopathy, and it has been shown that miR-15b is involved in exacerbating diabetic retinopathy by regulating *VEGF* expression. hsa-miR-20b-5p, hsa-miR-27b-3p, and hsa-miR-451a are also effective in causing this disease [12]. Strafella et al. also showed that their target miR-31, miR-23A, miR-27A, miR-20A, and miR-150 and *CFH*, *ARMS2*, *IL-8*, *TIMP3*, and *SLC16A8* were involved as risk factors for AMD [13]. Glaucoma has also been shown to have hsa-miR-184, hsa-miR-486-5p, and hsa-miR-93-5p significant expression and activity in patients with glaucoma (Y. [14]). miRNAs play an essential role in diagnosing and treating diabetic retinopathy, such as miR-20a-5p, miR-20a-3p, miR-20b, miR-106a-5p, miR-27a-5p, miR-27b-3p, miR-206-3p, and miR-381-3p [15] and miR-20a-5p, miR-20b-5p, miR-27a-3p, miR-27b-3p, miR-206-3p, and miR-381-3p which are related to VEGF and TGFB and expressed in hypoxia [16]. High glucose also increases retinal neovascularization, which can cause irreversible damage to photoreceptors. In this regard, miR-20a-3p, miR-20a-5p, miR-106a-5p, and miR-20b are related to this phenomenon [17], but miR-204 function has not yet been thoroughly evaluated in retinopathies. This study was aimed at evaluating the function of miR-204 target genes in the development of glaucoma, retinoblastoma, and AMD. In this regard, miR-204 target genes were chosen, and the most appropriate datasets for the three retinopathies were selected. By performing continuous bioinformatics analysis, key gene and protein production relating miR-204 and retinopathies was identified to realize a better understanding of miR-204 performance in the retina.

## 2. Methods and Materials

### 2.1. Gene Datasets

In this study, the GEO database (https://www.ncbi.nlm.nih.gov/geo/) was used to select appropriate datasets. The GSE50195 dataset related to AMD disease examined 16 samples. The GSE27276 dataset related to glaucoma included 36 samples. The GSE97508 dataset related to retinoblastoma disease had 9 samples (6 retinoblastoma samples and 3 control samples).

### 2.2. Preparation of Data for Bioinformatics Analysis

At this stage, the genes related to the three datasets of different retinopathies were extracted and stored in an Excel file using the GEO2R tool. Then, we separated the up- and downregulated genes and prepared cluster genes for future analysis. Genes were classified based on *p* value < 0.05 and logFC > 1.

### 2.3. Investigation of miR-204 Target Genes

The TargetScan (http://www.targetscan.org/vert_72/) database was used to examine miR-204 target genes, and then, the miRTargetLink database (https://ccb-web.cs.uni-saarland.de/mirtargetlink/) was used to plot the target genes' network. These two databases accurately separate their target genes and have comprehensive information to investigate the number of genes involved in various organs and tissues of the human body, the target genes have been uploaded to the Enrichr database (http://amp.pharm.mssm.edu/Enrichr/), and from the ontology section, we chose genes in the retina from the GENESEN TISSUE library and identified important signaling pathways through evaluating the PANTHER database (http://pantherdb.org/), because the PANTHER database has the ability to categorize data from a large to small scale, on the basis of cluster genes.

### 2.4. Shared Genes

After identification of the up- and downregulated retinopathies' genes, we selected miR-204 target genes using the TargetScan database. Then, we plotted the three retinopathies and miR-204 target genes and extracted their common ones.

### 2.5. Gene Ontology and Signaling Pathway Analysis

The genes related to miR-204 and retinopathies were separately uploaded to the Enrichr database, and important signaling pathways were extracted using the KEGG library (https://www.genome.jp/kegg/). Also, the Enrichr database was used to evaluate Gene Ontology (GO) from biological process libraries and molecular functions. After that, the genes were inserted into the ShinyGO database and hierarchical clusters and biological processes for the up- and downregulated genes were plotted to plot the GO diagrams. This database has more attractive figures than other databases in drawing the communication network between gene ontologies or other bioinformatics data.

### 2.6. Investigation of Cancer-Related Genes

The Gene Expression Profiling Interactive Analysis (GEPIA) database (http://gepia.cancer-pku.cn/) was used to evaluate the expression of genes selected for retinoblastoma. Survival plot, gene expression levels, and stage plots between selected genes and common cancers were studied and designed.

 

## 3. Results

### 3.1. Signaling Pathways Associated with miR-204 Target Genes in the Retina

**Figure 1:**
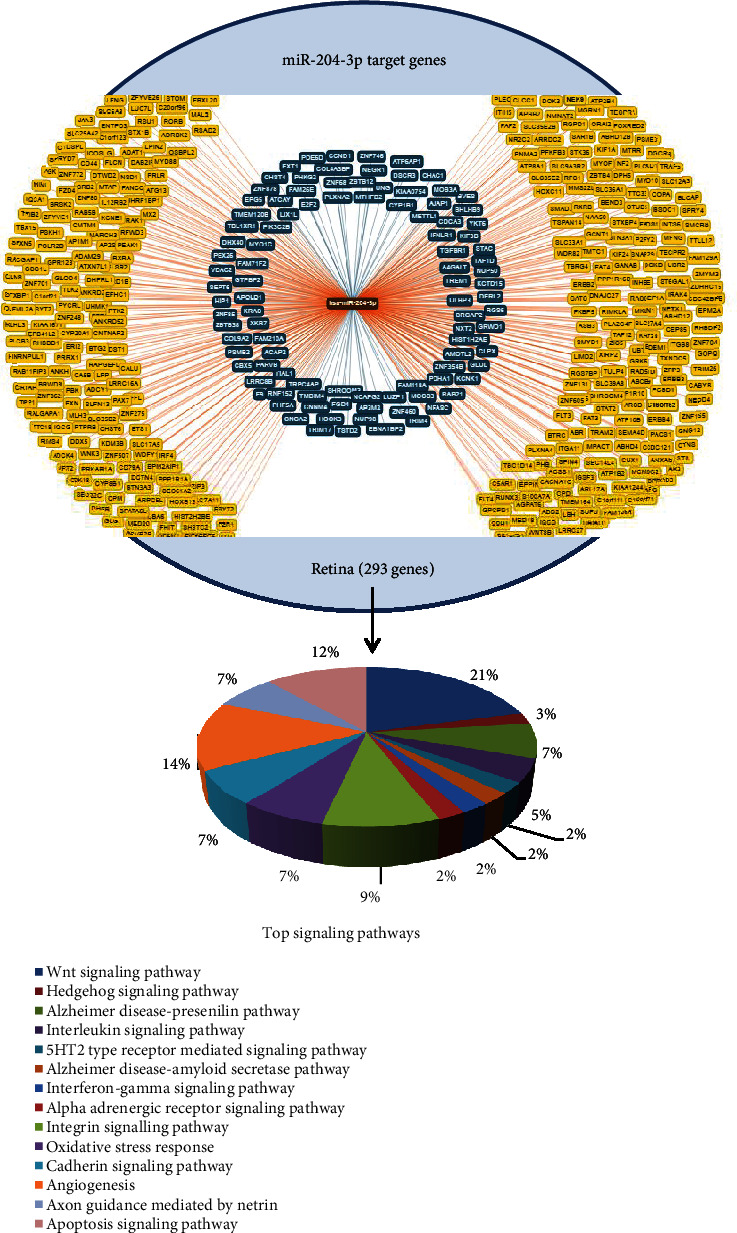
miR-204 target genes are plotted. Among the 792 target genes, 293 genes were present in the retina involved in many critical molecular pathways.

In the present study, 792 genes were directly predicted as miR-204 target genes. Of these, 293 genes were found in the retina. These 293 genes are involved in important signaling pathways such as apoptosis, Wnt, hedgehog, Alzheimer's disease pathway, interleukin, angiogenesis, and axon guidance signaling pathway (Figure 1).

### 3.2. NGF Signaling Pathways, Junctions, and Cell Division Were Significantly Associated with miR-204 Target Genes in Glaucoma, Retinoblastoma, and AMD

**Figure 2:**
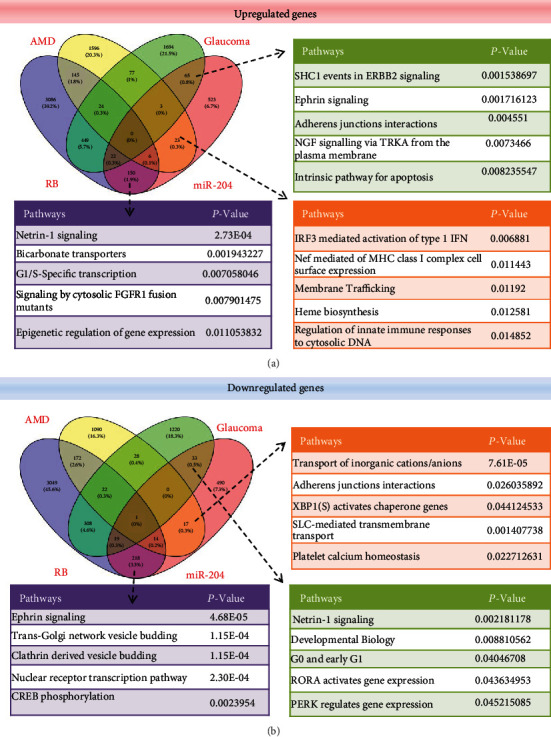
Gene expression profiles identify the similarity between miR-204 target genes in glaucoma, retinoblastoma, and AMD, and their important signal pathways are listed in the table: (a) upregulated genes and (b) downregulated genes.

Based on the Venny diagram, miR-204 and three retinopathies of glaucoma, retinoblastoma, and AMD shared 238 upregulated and 276 downregulated genes. The SHC1 events in ERBB2, adherent junctions' interactions, NGF signaling via TRKA from the plasma membrane, IRF3-mediated activation of type 1 IFN, Nef-mediated MHC class I complex cell surface expression, heme biosynthesis, netrin-1 signaling, and G1/S-specific transcription signaling pathways for the upregulated genes and G0 and early G1, RORA-activated gene expression, PERK-regulated gene expression, transport of inorganic cations/anions, adherent junctions' interactions, CREB phosphorylation, and ephrin signaling for the downregulated genes were shared by the three retinopathies and miR-204 (Figure 2).

### 3.3. Enrichment Analysis of Biological Processes

**Figure 3:**
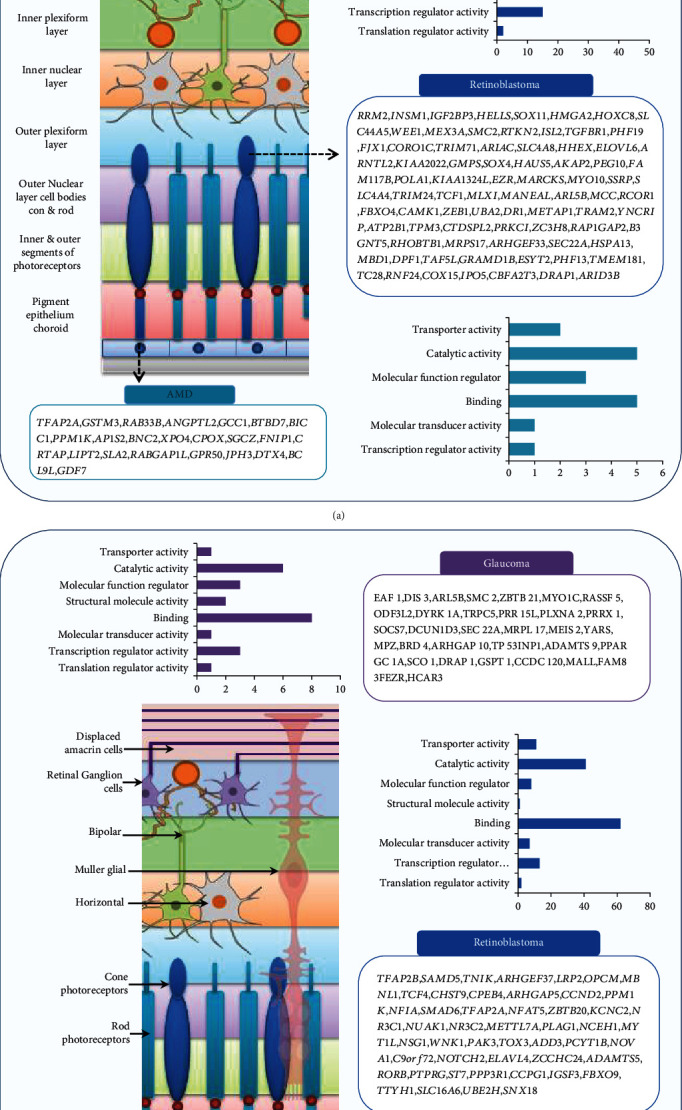
The molecular functions between the miR-204 target genes, glaucoma, retinoblastoma, and AMD. An arrow indicates the main origin of each retinopathy and its cells: (a) upregulated genes and (b) downregulated genes.

**Figure 4:**
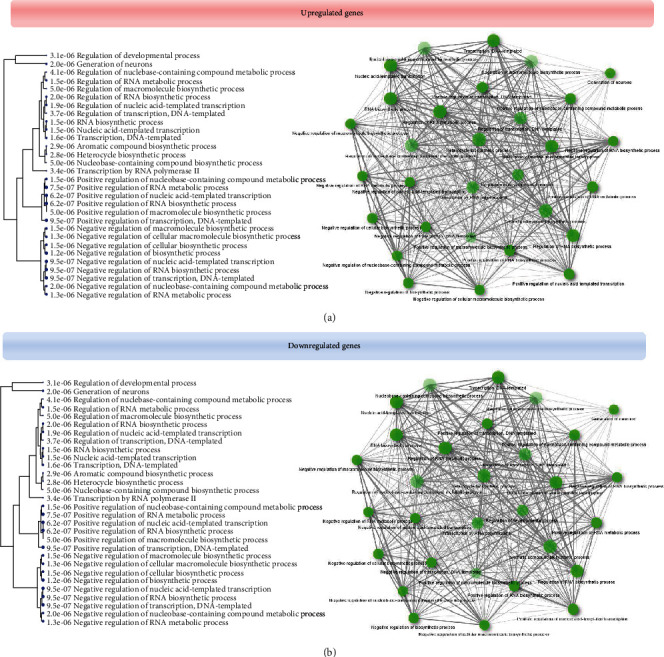
The biological processes and hierarchical clusters between the shared genes and miR-204 and retinopathies: (a) upregulated genes and (b) downregulated genes.

The genes shared by miR-204 and retinopathies were pooled, and biological processes of noradrenergic neuron differentiation, nervous system development, sympathetic nervous system development, cellular senescence regulation, chromatin remodeling, transmembrane receptor protein serine/threonine kinase signaling pathway regulation, and processes such as regulation of cellular response to growth factor stimulus, actin filament organization, tube morphogenesis, and intracellular transport for upregulated genes and neuron projection morphogenesis, generation of neurons, endocytosis, negative regulation of transcription by RNA polymerase II, negative regulation of cellular protein metabolic process, regulation of cell differentiation, and regulation of intracellular signal transduction for downregulated genes were confirmed (Figures 3 and 4 and Table 1).

### 3.4. Candidate Genes between Retinoblastoma and miR-204

**Figure 5:**
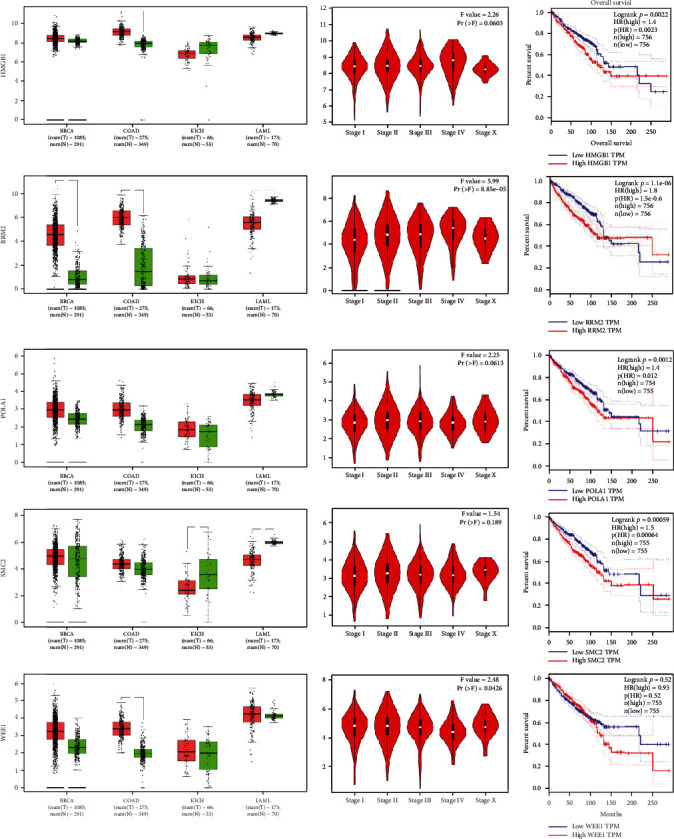
Important genes (*HMGB1*, *RRM2, POLA1*, *SMC2*, and *WEE1*) associated with tumorigenesis and retinoblastoma progression are identified. The genes were compared with four cancers: leukemia, colon cancer, kidney cancer, and breast cancer, and their expression was shown as a box plot. As shown in the figure, all genes are continuously expressed in the major stages of cancer. Survival charts also show that over time, mortality in individuals rises to less than 50 percent.

In this study, 150 upregulated and 216 downregulated target genes of miR-204 were selected, which are shared by retinoblastoma. The STRING database plotted their protein network, and the five proteins WEE1, SMC2, HMGB1, RRM2, and POLA1 that were most associated with the range of activity involved in tumorigenesis and retinoblastoma progression were selected from 366 genes. The modeled protein network had 38 nodes and 17 edges with a PPI enrichment *p* value of 0.000101 (protein network not shown) (Figure 5).

## 4. Discussion

miR-204 can be considered one of the most valuable miRNAs involved in retinal development and maintenance [18]. For this reason, disruption of this miRNA might cause a variety of retinopathies. For example, miR-204 plays a significant role in retinoblastoma and acts as a tumor suppressor, while it has much less expression in patients with retinoblastoma (X. [19]). Cyclin-D2 and MMP-9 are two key genes that are regulated by miR-204 in retinoblastoma. High expression of cyclin-D2 and MMP-9 increases the cell division rate and progression of retinoblastoma [19]. On the other hand, expression of miR-204 in glaucoma and optic nerve injury is high, which by disrupting GAP-43 disrupts the MyD88/TLR4/NFKB pathway and ultimately kills retinal cells (N. [20]). miR-204 also alters the extracellular matrix by acting on TGF-B through Let-7a which is associated with abnormalities in the retina structure (N. [20]). In AMD, the role of miR-204 is unique and affects LAMP1 by regulating EZR gene expression, which is influential in the formation of phagocytic vesicles. Interruption of this pathway meddles with the biological processes dependent on the phagocytic vesicles of RPE cells [21].

In this study, signaling pathways were analyzed by a bioinformatics approach towards miR-204 target genes, and other related pathways were identified in the three retinopathies of glaucoma, retinoblastoma, and AMD. Ephrin, NGF, and ERBB2 as three important pathways identified by their high expression are shared by glaucoma and miR-204 target genes. In a study by Xu et al., which was done in a mouse model of chronic ocular hypertension (COH) to damage optic nerves and ganglion cells, it was shown that ephrin A3 and ephrin A4 increased the expression and accelerate the Glu A2 pathway, which impaired calcium absorption and ions. Diminished calcium absorption generally impairs the function of ganglion cell dendrites and induces apoptosis [22]. Wang et al. showed that KLF16 plays a vital role in neuronal cell growth as a decisive transcription factor. To control KLF16, ephrinA5 has an effective regulatory role in this pathway to regulate retinal tissue and the function of the visual cycle [23]. Protein tyrosine kinase as a part of several cell surface receptor complexes apparently needs a coreceptor for ligand binding. GP30 is a potential ligand for this receptor and regulates outgrowth and stabilization of peripheral microtubules (MTs). Upon ERBB2 activation, the MEMO1-RHOA-DIAPH1 signaling pathway elicits phosphorylation and thus inhibition of GSK3B at the cell membrane. A study by Chen et al. on glaucoma patients and healthy individuals, using bioinformatics analysis, introduced several key genes that were for the first time nominated as associated with glaucoma progression. They measured the expression of genes on tissue samples of patients and healthy individuals and confirmed genes [24]. Another study by Kwon et al. showed that myocilin, a glaucoma-related gene, binds to ERBB2 receptors and activates downstream PI3K/Akt pathways involved in the maintenance and development of nerve cells and neuronal myelination. Defects in this pathway naturally disrupt neural signals and the network between neurons [25]. Nerve growth factors also play an important role in strengthening the nervous system as well as the retina. It has been reported that the expression of two important neurological factors BDNF and NGF in glaucoma patients was significantly reduced compared to healthy individuals; such reductions can lead to poor cell function and impaired nervous growth and development [26, 27]. In another study, Liu et al. applied more hydrostatic pressure to mice with damaged ganglion cells. Increased hydrostatic pressure induced apoptosis and decreased ganglion cell viability due to disruption of the NGF/AKT/CREB signal pathway (H. [28]).

Netrin-1 is one of the most important genes that play an essential role in axon guidance and cell formation and connections. In a study conducted by Chehrazi et al., it was indicated that the expression pattern was decreased in the damaged optic nerve model compared to the healthy sample in mice. Low expression of netrin-1 can interfere with the growth and integration of nerve cells as well as ganglion cells in the retina [29]. Moreover, it was found that netrin-1 is involved in retinal neovascularization and is highly expressed during neovascularization. Then, using lentivirus, we inserted shRNA into a mouse model and observed inhibition of the activity of netrin-1. Subsequent evaluations revealed that decreased netrin-1 expression reduced neovascularization in retinal tissue [30]. PERK is a stress-sensitive metabolic gene that operates in the endoplasmic reticulum. In a mouse model of glaucoma, it was found that increased stress in the endoplasmic reticulum increased apoptosis in ganglion cells, and PERK played an important role in inducing apoptosis and stress exacerbation [31]. Wang et al. in a similar study obtained similar results on the concurrent trabecular bone (Y. [32]).

In AMD, three pathways for heme synthesis, IRF3, and cellular connections were identified. The study by Shetty et al. showed that heme synthesis plays a very important role in angiogenesis and nutrition transport at the tissue and microenvironment levels. Heme synthesis in vessel-rich tissues, such as the choroid, can play an imperative role in improving function and healing. Besides, because choroid is closely related to RPE cells, disruption of this pathway may play a role in AMD development [33]. IRF3, an interferon regulatory factor 3, plays a vital role in the innate immune system and the response to cell death. By using siRNAs against TLR and IRF3 in AMD disease, TLR induced apoptosis and retinal degeneration by initiating the caspase pathway, but this was not confirmed in IRF3 [34]. But in Wu et al.'s study, it was found that the CGAS/STING signaling pathway which is sensitive to DNA damage is activated and triggers several inflammatory factors, including cytokines and IRF3, contributing to AMD progress [35]. Adhesion junctions have always been among the most critical factors in integrating cells to create the desired tissue. Therefore, in a study by Joseph et al. on retinal cell connections with a focus on cellular aging, it was shown that S1P is a regulator of cellular junctions and is very effective in amplifying N-cadherin in RPE, choroid, and Muller glial cells. However, a 15-month-old mouse model study found that S1P was impaired and could play a role in retinal tissue degeneration [36]. The study by Narimatsu et al. showed that exposing mice to light continuously and over time disrupted actin cytoskeletons in the blood retinal barrier and RPE cells by activating reactive oxygen species and Rho/ROCK pathways [37].

Retinoblastoma is a cancerous disease; for its activity, retinoblastoma needs to use miRNAs to regulate the expression of genes suitable for tumorigenesis and invasion to other organs. In a study by Fernandez et al. done on the Y79 cell line, it was found that FGF-1 and FGFR-1 were highly expressed and played an important role in cell division through heparan sulfate and proteoglycans [38]. As discussed above, netrin-1 is involved not only in glaucoma but also in cancer. In Shekarabi et al.'s study, it was found that high expression of netrin-1 can trigger important cytoskeletal factors such as cdc42, Rho, Rac1, Pak1, and N-WAS, which play a significant role in cell division and axonal guidance [39]. Galardi et al. performed another intriguing study on proteomics of exosomes derived from tumor particles present in the vitreous. Netrin-1 showed a high expression in retinoblastoma, which could confirm its carcinogenic role. Netrin-1 also inhibited P53 by acting on UCN5B [40]. Another pathway selected in retinoblastoma was clathrin. L1 acts as a transmembrane glycoprotein that promotes drug resistance in retinoblastoma and promotes invasion and cell division. Kamiguchi et al. showed that L1 binds to AP 2 and causes clathrin-dependent endocytosis [41]. CREB, as a necessary regulatory factor, is associated with three genes, cyclin D1, cdk4, and RB1. If overexpressed, CREB can reduce cell division, progression, and invasion of cancer [42].

As shown in the protein network plot for retinoblastoma-related genes, SMC2, HMGB1, WEE1, RRM2, and POLA1 proteins showed an association with other proteins. HMGB1 plays a very important role in division and invasion of cancer cells. Therefore, several studies have been performed to inhibit the function of HMGB1 by siRNAs and miRNAs, and all have shown that inhibition of HMGB1 increases the induction of apoptosis and decreases cell division, progression, and invasion of Y79 cells [23, 43, 44]. Limited studies have examined PolA1 in retinoblastoma. A study by Ganguly et al. performed on retinoblastoma and healthy individuals, using bioinformatics analysis, showed that PolA1 was influential in retinoblastoma disease progression [45]. But other studies have shown the carcinogenic activity of PolA1 in bladder [46] and liver cancer [47]. RRM2 has a significant activity in DNA synthesis and is effective in inhibiting the Wnt signaling pathway. There are also limited studies on the cancerous role of this gene in retinoblastoma. Two studies similarly examined retinoblastoma gene expression profiles in healthy individuals and finally showed that RRM2 can play an important role in cell division and its different phases [48, 49]. SMC2 plays an essential role in chromosome densification and the formation of supercoils. Rajasekaran et al.'s study showed that SMC2 was significantly more pronounced in retinoblastoma patients than in healthy individuals, but the exact mode of action of this protein in retinoblastoma is still unknown [50]. Disruption of the DNA damage response pathway can be the beginning of tumorigenesis. WEE1 is a gene that plays a vital role in response to DNA damage and the G2/M phase of the cell cycle. Disruption of WEE1 has been investigated in various cancers, and various inhibitors have been used as a treatment through WEE1 regulation, which has yielded good results [51, 52].

Wu et al. showed that, among the genes that were nominated by bioinformatics analysis of the GSE97508 dataset, *TRIM59* was significantly upregulated in three retinoblastoma cell lines compared with control; this gene plays an oncogenic role in retinoblastoma by activating the p38-MAPK signaling pathway (C. [53]). It has been reported that *CLUL1*, *CNGB1*, *ROM1*, *LRRC39*, and *RDH12* genes in different retinoblastoma subtypes are involved in the progression and development of the disease, which can be useful as biomarkers (M. [54]). Also, the study of Zeng et al. demonstrated that bioinformatics analysis of the GSE97508 dataset, with the approach of examining epigenetic factors in retinoblastoma, can create significant diagnostic or therapeutic methods. *TTK*, *RRM2*, and *CDK1* were selected as candidates [55]. In the present study, *RRM2* was also selected as a candidate gene related to miR-204.

Studies have also been done for the GSE27276 dataset. Most of these studies selected gene expression profiles in glaucoma and trabecular mutilation, and in each of these data-related analyzes, different genes, such as *LCN2*, *MAOA*, *HBB*, *PAX6*, *FN1*, and *CREB1* [56] and *COL4A4*, *COL3A1*, *COL1A2*, *ITGB5*, *COL5A2*, and *COL5A1* [57], and collagen, actin, and cell-matrix interactions were examined. Inflammation included pathways, entailing NF-*κ*B and arachidonic acid [58], which have been nominated to find more accurate molecular events.

In the AMD-related dataset (GSE50195), a study by Zhao et al. indicated that between AMD and healthy retinal tissue, *TNC*, *GRP*, *TRAF6*, *ADAMTS5*, *GPX3*, *FAP*, *DHCR7*, and *FDFT1* genes acted as upstream factors for pathogenesis in AMD [59]. An intriguing survey by Ashikawa et al. showed that early detection in the early stages of AMD is involved in the recovery of these patients. Thus, in its AMD zebrafish model, RPE-choroid tissue was isolated, and PCR was performed. Bioinformatics analysis revealed that *FADS2* and *ACAT2* are involved in disease progression through activation of sterol regulatory proteins. By knocking down the genes and examining the pathways, they showed that the diagnosis and examination of *FADS2* and *ACAT2* in the early stages of the disease will help to improve the quality of treatments [60]. Zhang et al. displayed that RHO, PDE6A, 3′,5′-cyclic-GMP phosphodiesterase, and G protein alpha pathways play a role in the development and intensification of AMD [61]. Also, the study by Su et al., which analyzed this database, eventually nominated hsa_circRNA7329/hsa-miR-9/SCD, where hsa_circRNA7329 is in line with AMD's development [62]. Each of the studies that have been done so far examined the gene expression profile in these three diseases separately and selected the markers for diagnosis or treatment with different approaches. In the present study, we specifically evaluated miR-204 target genes in the retina and retinopathies. Therefore, mir-204 can act as a key miRNA in the retina and can use modern drug systems such as biodegradable microspheres as intravitreal delivery systems for prolonged drug release [63, 64]. Therefore, appropriate antagomir and agomir can be used as pharmacological approaches to treat various retinopathies associated with miR-204.

## 5. Conclusion

Finally, miR-204 acts as a key miRNA in the retina and induces and regulates various pathways such as the growth and development of nerve cells, immune system stimulation, and tumorigenesis. miR-204 target genes were also higher in retinoblastoma, followed by other target genes in glaucoma and macular degeneration. Approximately one-third of the miR-204 target genes are present in the retina, indicating that the miR-204 can affect multiple pathways in the retina. This study examined some of these signaling pathways and their genes and proteins. However, further studies are needed to investigate this miR-204 mechanism in the retina in the future.

## Figures and Tables

**Table 1:** Candidate genes between retinoblastoma, glaucoma, and AMD with miR-204.

Genes	LogFC	*p* value
Retinoblastoma		
WEE1	3.10562667	8.76*E* − 06
SMC2	2.98285333	9.97*E* − 06
HMGB1	0.656	6.90*E* − 03
RRM2	8.80672667	1.72*E* − 10
POLA1	1.59243333	4.94*E* − 04
AMD		
SLC24A2	-0.66397667	0.00775204
DTX4	0.20969803	0.02179582
Glaucoma		
EPHB6	0.17718077	4.94*E* − 03
EFNB3	0.20454188	1.32*E* − 04
SHC1	0.53068987	1.39*E* − 02

## Data Availability

In this study, the GEO database (https://www.ncbi.nlm.nih.gov/geo/) was used to select appropriate datasets. The GSE50195 dataset related to AMD disease examined 16 samples. The GSE27276 dataset related to glaucoma included 36 samples. The GSE97508 dataset related to retinoblastoma disease was 9 samples (6 retinoblastoma samples and 3 control samples).

## Supplementary Materials

Supplementary Materials To better understand bioinformatics analysis, the schematic pathway of the study is shown in Figure S1.
