# Supplementary material for: Investigation of Key Signaling Pathways Associating miR-204 and Common Retinopathies
Source: Biomed Res Int. 2021 Oct 4;2021:5568113. doi: 10.1155/2021/5568113 (PMC8505061; doi:10.1155/2021/5568113)
Supplement: Supplementary Materials — To better understand bioinformatics analysis, the schematic pathway of the study is shown in Figure S1. [file 5568113.f1.docx]

**Supplementary Information**

**Figure S1:** Schematic pathway of bioinformatics analysis, GEO database was used to study the gene expression profile in common retinopathies. We also used the Targetscan database to view miR-204 target genes.
